# Clustering in surgical trials - database of intracluster correlations

**DOI:** 10.1186/1745-6215-13-2

**Published:** 2012-01-04

**Authors:** Jonathan A Cook, Thomas Bruckner, Graeme S MacLennan, Christoph M Seiler

**Affiliations:** 1 Health Services Research Unit, University of Aberdeen, 3rd Floor, Health Sciences Building, Foresterhill, Aberdeen, AB25 2ZD, UK; 2 Institute of Medical Biometry and Informatics, University of Heidelberg, Germany; 3 Department of General, Visceral and Trauma Surgery, University of Heidelberg, Germany

**Keywords:** Surgery, ICC, multicentre, clustering

## Abstract

**Background:**

Randomised trials evaluation of surgical interventions are often designed and analysed as if the outcome of individual patients is independent of the surgeon providing the intervention. There is reason to expect outcomes for patients treated by the same surgeon tend to be more similar than those under the care of another surgeon due to previous experience, individual practice, training, and infrastructure. Such a phenomenon is referred to as the clustering effect and potentially impacts on the design and analysis adopted and thereby the required sample size. The aim of this work was to inform trial design by quantifying clustering effects (at both centre and surgeon level) for various outcomes using a database of surgical trials.

**Methods:**

Intracluster correlation coefficients (ICCs) were calculated for outcomes from a set of 10 multicentre surgical trials for a range of outcomes and different time points for clustering at both the centre and surgeon level.

**Results:**

ICCs were calculated for 198 outcomes across the 10 trials at both centre and surgeon cluster levels. The number of cases varied from 138 to 1370 across the trials. The median (range) average cluster size was 32 (9 to 51) and 6 (3 to 30) for centre and surgeon levels respectively. ICC estimates varied substantially between outcome type though uncertainty around individual ICC estimates was substantial, which was reflected in generally wide confidence intervals.

**Conclusions:**

This database of surgical trials provides trialists with valuable information on how to design surgical trials. Our data suggests clustering of outcome is more of an issue than has been previously acknowledged. We anticipate that over time the addition of ICCs from further surgical trial datasets to our database will further inform the design of surgical trials.

## Background

Patients under the care of the same surgeon will be influenced in a similar manner due to the surgeon's practice, skill and experience [1]. Outcomes for those treated by the same surgeon tend to be more similar than those under the care of another surgeon due to previous experience, individual practice, training, and infrastructure [2]. This phenomenon is referred to as the clustering effect. While the impact of clustering of outcome has been widely acknowledged for cluster randomised controlled (C-RCTs) trials for some time [1,3,4], its potential impact upon individually randomised controlled trials (RCTs) evaluating therapist dependent interventions, such as surgical interventions, has only been highlighted more recently [1,5]. Models which allow for clustering have been used to analyse surgical trials though this is not commonly done [2,6-8].

Clustering has implications for the required sample size of a RCT; the impact depends upon the design and analysis adopted. For example, a RCT comparing two surgical interventions which adopts an expertise-based trial design, where each participating surgeon delivers only one of the two surgical interventions under evaluation, clustering is incorporated into the design at the surgeon-level in a similar manner to a C-RCT [9]. Surgical versus medical trials (e.g. laparoscopic surgery versus medical management [10]) have naturally been conducted using an expertise-based design where relevant health professional only deliver one or the other of the interventions [11]. Such a design, other factors being equal, potentially leads to a relative loss of precision and increase in the required sample size. In contrast, the adoption of a stratified within-surgeon design can lead to a reduction in the sample size [12]. A further trial design option is a hybrid of these two approaches, such as a *surgeon preference trial*, where each participating surgeon opts to deliver either one of the two interventions or both. A variety of statistical methods which allow for clustering are available including both fixed and random effects approaches [13].

Recruitment of participants to a RCT across multiple centres (multicentre RCT) is commonly adopted to increase both generalisability and the rate of recruitment. Similar reasons, even if only implicitly recognised, lead to the participation of multiple surgeons within and across centres. Clustering in multicentre surgical trials, as with other therapist dependent trials, could in principle, address clustering at the centre and/or surgeon (therapist) level. A design consideration is whether randomisation and the analysis should account for clustering at the centre and/or surgeon in a multi-centre surgical RCT.

The statistical measure of the clustering between participants under the care of a surgeon or centre is known as the *intracluster correlation coefficient *[14], or *ICC*. The ICC can be defined as the proportion of the total variation in the participant outcome that can be attributed to the difference between clusters (e.g. surgeon) and is often represented by ρ. The magnitude of clustering could be influenced by a number of factors such as cluster type (e.g. centre), setting and type of outcome and the time since receiving the intervention [15].

Where a clustering effect exists, this has direct implications for the sample size calculations and the statistical analysis that is required. Standard sample size calculations and analysis techniques assume that the outcome for individual participants will be independent and consequently they will incorrectly estimate (typically underestimate) the true sample size required to detect a pre-specified difference with the desired precision and power. Correspondingly, statistical analyses which ignore the presence of clustering will likely result in overly precise, and potentially misleading, results. Whereas the impact is typically of an inflation in the sample size in the case of C-RCTs, for individually randomised trials the required sample size may be reduced [12].

Trialists have little data upon which to assess the impact of clustering and appropriately modify trial design. Quantifying the clustering effect would aid the design of surgical trials [4]. There is, however, little information available on the likely magnitude of ICCs in surgical trials and it is very rare for surgical trials to use such estimates during the design stage though there is a growing awareness of the need to do so [7,9]. The aim of this work was to inform trial design by quantifying clustering effects (at both centre and surgeon level) for various outcomes using a database of surgical trials.

## Methods

ICCs were calculated for outcomes from a set of 10 multicentre surgical trials for a range of outcomes and different time points (where applicable). Clustering was assessed at both the centre and surgeon level independently of each other. Trials recruited participants from centres across the UK and Ireland, Germany or Europe. Interventions under evaluation included general (abdominal, endocrine, pancreatic and upper gastrointestinal), ophthalmology and orthopaedic (hip and knee) surgical specialties. Of the 10 trials, five each included centre and surgeon respectively in the randomisation algorithm. One study [10] which evaluated a surgical versus medical comparison had an expertise-based trial design. Trials varied in size from 138 to 1370 participants; the median (range) number of centres and surgeon were 19 (8, 27) and 49 (16, 191) respectively. Outcomes evaluated included perioperative (e.g. operation time), surgical (e.g. length of stay and recurrence of hernia), functional (e.g. visual function) and both overall (e.g. EQ-5D and SF-36) and disease-specific (e.g. Oxford knee score) measures. The length of follow-up available varied from short-term (six months or less) to long-term (five years).

The ANOVA method was used to estimate an outcome's ICC along with bootstrapped 95% confidence intervals (CI)[16,17]; this was done separately for each trial. Two ANOVA models were used for every outcome; one where centre and one where surgeon was the clustering factor. These analyses were carried out in Stata 11.1 utilising in combination the bootstrap and loneway commands [18]. The bootstrap process allowed for the clustered nature of the data and 1000 replications were sampled. Both Bias Corrected (BC) and Bias Corrected and Accelerated (BCA) 95% Bootstrapped CIs were calculated for each outcome. If these bootstrapped CIs were not calculable, a CI based upon the percentile bootstrap method was used for the ICC. The operating surgeon was used to define the cluster if surgeon was not included in the randomisation algorithm. Post-intervention data from the surgical interventions arms were used to calculated the ICCs without adjustment for treatment. Clustering information (cluster size distribution and outcome prevalence/mean) were generated [19].

The design effect (or variance inflation factor) is the value which the standard sample size needs to be multiplied by to account for the impact of clustering. For a continuous outcome the impact upon a stratified within-surgeon design has been shown to be 1-ρ reflecting a potential reduction in sample size over a standard analysis [12]. Under an expertise-based trial, the formula 1+(average cluster size-1)*ρ can be used reflecting the need to inflate the size to compensate for loss of information. To illustrate the possible impact of adopting an expertise-based trial design or stratified (or minimised) within-surgeon design using the data from the 10 trials to present plausible scenarios for two common outcomes - one surgical (operation time) and one patient-reported (EQ-5D at 12 months). In addition to the actual cluster sizes, an adjusted cluster size using the formula (∑n_i_^2^)/∑n_i_) was also used which allows for the impact of the variation in cluster size to be taken into account [20].

Exploration of the relative contribution of the three levels (1. participant, 2. surgeon and 3. centre) to the overall variance was carried out using a three level model (xtmixed command in Stata) for the EQ-5D at 12 months. The three corresponding ICCs (Level 2 ICC, Level 3 ICC and Levels 2 and 3 ICC) for this model were calculated along with BCA 95% CIs.

## Results

Details of the 10 trials and information on the cluster sizes are reported in Table 1. The median (range) average cluster size was 32 (9 to 51) and 6 (3 to 30) for centre and surgeon levels respectively. Surgeon cluster size was smaller than centre size as expected for surgeons nested within centres.

**Table 1:** Surgical trial datasets

Trial	Surgical intervention(s)	N	No. Outcomes	No. Surgeon	No. Centres
CLIVIT [26]	Thyroid surgery with clips or ligatures	491	5	125	13
DISPACT [27]	Distal pancreatectomy with stapler or hand-sewn closure	352	6	126	21
FILMS [28]	Macular hole surgery with/without peeling of Intra-limiting membrane.	138	19	N/A	9
HERNIA [29]	Open vs. laparoscopic Inguinal hernia repair	928	41	31	24
INSECT [30]	Midline laparotomy with one of three closure methods	625	6	191	24
KATMETAL [6]	Total knee arthroplasty with/without metal backed tibial component	409	26	16	8
KATMOBILE [6]	Total knee arthroplasty with/without mobile bearing between tibial and femoral components	539	26	24	13
KATPATELLA [6]	Total knee arthroplasty with/without patella resurfacing	1370	26	99	27
RELFUX [10]	Laparoscopic fundoplication	178	20	31	19
STARS [31]	Reduction and fixation, bipolar hemiarthroplasty or total hip arthroplasty	298	23	49	11

ICCs were calculated for 198 difference outcomes across the 10 trials at both centre and surgeon cluster levels. For 21 outcomes it was not possible to calculate bias corrected bootstrapped CIs at centre and/or surgeon level and a CI based upon the bootstrap percentile method was used instead. ICC estimates and corresponding CIs of 48 outcomes (selected based upon primary outcomes of the included trials and other commonly reported outcomes in the surgical literature) are given in Table 2. Full details are available online at http://www.abdn.ac.uk/hsru/research/research-tools/study-design.

**Table 2:** Individual ICC estimates at centre and surgeon levels

		Centre level clustering							Surgeon level clustering						
*Outcome*	*Trial*	*N*	*Mean/Prop*	*ICC*	*BC 95% CI*		*BCA 95% CI*		*N*	*Mean/Prop*	*ICC*	*BC 95% CI*		*BCA 95% CI*	
EQ-5D 1 wk	HERNIA	610	0.71	0.029	(0.003,	0.086)	(0.004,	0.086)	610	0.71	0.023	(0.003,	0.057)	(0.004,	0.070)
EQ-5D 3 m	HERNIA	497	0.86	0	(0.000,	0.000)*	(.,	.)	497	0.86	0	(0.000,	0.000)*	(.,	.)
EQ-5D 3 m	KATMETAL	361	0.66	0	(0.000,	0.000)*	(.,	.)	361	0.66	0.014	(0.000,	0.104)	(0.000,	0.119)
EQ-5D 3 m	KATMOBILE	448	0.66	0.06	(0.005,	0.133)	(0.007,	0.140)	448	0.66	0.071	(0.021,	0.149)	(0.023,	0.159)
EQ-5D 3 m	KATPATELLA	1169	0.70	0.008	(0.000,	0.041)	(0.000,	0.047)	1169	0.70	0.005	(0.000,	0.032)	(0.000,	0.032)
EQ-5D 3 m	REFLUX	149	0.79	0.001	(0.000,	0.120)	(0.000,	0.105)	104	0.82	0.001	(0.000,	0.120)	(0.000,	0.105)
EQ-5D 4 m	STARS	277	0.61	0	(0.000,	0.043)	(0.000,	0.043)	277	0.61	0.015	(0.000,	0.109)	(0.000,	0.112)
EQ-5D 12 m	KATMETAL	354	0.71	0.006	(0.000,	0.070)	(0.000,	0.070)	354	0.71	0	(0.000,	0.025)	(0.000,	0.022)
EQ-5D 12 m	KATMOBILE	448	0.70	0.040	(0.004,	0.103)	(0.006,	0.107)	448	0.70	0.024	(0.000,	0.080)	(0.000,	0.094)
EQ-5D 12 m	KATPATELLA	1157	0.74	0.017	(0.000,	0.052)	(0.000,	0.061)	1157	0.74	0.040	(0.011,	0.076)	(0.013,	0.081)
EQ-5D 12 m	REFLUX	152	0.75	0.007	(0.000,	0.107)	(0.000,	0.100)	100	0.78	0.002	(0.000,	0.322)	(0.000,	0.285)
EQ-5D 12 m	STARS	274	0.63	0	(0.000,	0.063)	(.,	.)	274	0.63	0	(0.000,	0.083)	(.,	.)
EQ-5D 60 m	KATMETAL	302	0.68	0	(0.000,	0.000)*	(.,	.)	302	0.68	0	(0.000,	0.000)*	(.,	.)
EQ-5D 60 m	KATMOBILE	381	0.69	0.015	(0.000,	0.080)	(0.000,	0.089)	381	0.69	0.019	(0.000,	0.065)	(0.000,	0.076)
EQ-5D 60 m	KATPATELLA	997	0.71	0.002	(0.000,	0.021)	(0.000,	0.022)	997	0.71	0.009	(0.000,	0.047)	(0.000,	0.050)
Oxford knee score 3 m	KATMETAL	327	30.14	0	(0.000,	0.000)*	(.,	.)	327	30.14	0.007	(0.000,	0.057)	(0.000,	0.059)
Oxford knee score 3 m	KATMOBILE	389	29.88	0.073	(0.016,	0.158)	(0.020,	0.165)	389	29.88	0.068	(0.018,	0.130)	(0.024,	0.142)
Oxford knee score 3 m	KATPATELLA	1057	30.89	0.041	(0.014,	0.087)	(0.016,	0.092)	1057	30.89	0.05	(0.021,	0.087)	(0.021,	0.087)
Oxford knee score 12 m	KATMETAL	311	33.71	0.021	(0.000,	0.042)	(0.000,	0.042)	311	33.71	0.056	(0.000,	0.130)	(0.001,	0.148)
Oxford knee score 12 m	KATMOBILE	387	32.99	0.063	(0.023,	0.134)	(0.026,	0.144)	387	32.99	0.059	(0.012,	0.134)	(0.014,	0.139)
Oxford knee score 12 m	KATPATELLA	1010	34.73	0.027	(0.004,	0.071)	(0.005,	0.076)	1010	34.73	0.047	(0.015,	0.094)	(0.016,	0.101)
Oxford knee score 60 m	KATMETAL	284	34.14	0	(0.000,	0.000)*	(.,	.)	284	34.14	0.002	(0.000,	0.021)	(0.000,	0.021)
Oxford knee score 60 m	KATMOBILE	350	33.43	0.044	(0.000,	0.123)	(0.000,	0.160)	350	33.43	0.051	(0.000,	0.121)	(0.004,	0.143)
Oxford knee score 60 m	KATPATELLA	928	34.90	0.045	(0.016,	0.086)	(0.017,	0.090)	928	34.90	0.037	(0.003,	0.074)	(0.005,	0.077)
Operating time (min)	CLIVIT	483	118.60	0.184	(0.084,	0.373)	(0.069,	0.333)	479	118.93	0.392	(0.244,	0.524)	(0.257,	0.528)
Operating time (min)	DISPACT	344	190.03	0.268	(0.188,	0.418)	(0.188,	0.418)	344	190.03	0.395	(0.254,	0.506)	(0.259,	0.508)
Operating time (min)	FILMS	125	67.55	0.212	(0.009,	0.451)	(0.049,	0.486)	NA						
Closure time (min)	INSECT	580	15.02	0.331	(0.129,	0.587)	(0.149,	0.661)	579	15.03	0.466	(0.338,	0.593)	(0.342,	0.600)
Operating time (min)	KATMETAL	398	106.70	0.449	(0.172,	0.726)	(0.183,	0.731)	398	106.70	0.514	(0.278,	0.727)	(0.302,	0.752)
Operating time (min)	KATMOBILE	503	122.37	0.167	(0.092,	0.294)	(0.093,	0.295)	503	122.37	0.199	(0.073,	0.390)	(0.073,	0.390)
Operating time (min)	KATPATELLA	1302	126.75	0.370	(0.254,	0.470)	(0.259,	0.478)	1302	126.75	0.445	(0.360,	0.524)	(0.369,	0.533)
Operating time (min)	REFLUX	108	112.81	0.375	(0.066,	0.623)	(0.060,	0.623)	104	113.16	0.375	(0.066,	0.623)	(0.060,	0.623)
Operating time (min)	STARS	298	62.40	0.072	(0.021,	0.278)	(0.022,	0.278)	298	62.40	0.093	(0.003,	0.222)	(0.013,	0.248)
Length of stay (days)	CLIVIT	488	3.05	0.065	(0.000,	0.367)	(0.000,	0.319)	485	3.05	0	(0.000,	0.397)	(0.000,	0.345)
Length of stay (days)	DISPACT	348	15.41	0.111	(0.012,	0.217)	(0.005,	0.210)	348	15.41	0.045	(0.000,	0.307)	(0.000,	0.266)
Length of stay (days)	INSECT	589	14.59	0.012	(0.000,	0.059)	(0.000,	0.049)	588	14.59	0	(0.000,	0.525)	(0.000,	0.421)
Length of stay (days)	REFLUX	108	2.38	0.345	(0.128,	0.590)	(0.146,	0.601)	104	2.38	0.345	(0.128,	0.590)	(0.146,	0.601)
Length of stay (days)	STARS	298	12.26	0.104	(0.002,	0.354)	(0.014,	0.420)	298	12.26	0.104	(0.002,	0.354)	(0.014,	0.420)
Fistula	DISPACT	352	0.29	0.122	(0.046,	0.301)	(0.039,	0.284)	352	0.29	0.084	(0.000,	0.220)	(0.000,	0.236)
Complication 6 m	FILMS	126	0.38	0	(0.000,	0.000)*	(.,	.)	NA						
Complication 1 wk	HERNIA	717	0.37	0	(0.000,	0.017)	(.,	.)	717	0.37	0.009	(0.000,	0.050)	(0.000,	0.053)
Wound infection	INSECT	625	0.16	0.01	(0.000,	0.037)	(0.000,	0.043)	610	0.16	0.072	(0.000,	0.183)	(0.000,	0.186)
HRQ overall score 4 m	STARS	276	68.85	0.058	(0.020,	0.207)	(0.019,	0.207)	276	68.85	0.114	(0.030,	0.222)	(0.032,	0.224)
HRQ overall score 12 m	STARS	258	74.04	0	(0.000,	0.093)	(0.000,	0.062)	258	74.04	0	(0.000,	0.126)	(.,	.)
HRQ overall score 24 m	STARS	236	75.37	0	(0.000,	0.000)*	(.,	.)	236	75.37	0	(0.000,	0.167)	(.,	.)
RQLS score 3 m	REFLUX	141	83.85	0.143	(0.000,	0.392)	(0.009,	0.449)	100	86.34	0.143	(0.000,	0.392)	(0.009,	0.449)
RQLS score 12 m	REFLUX	145	84.58	0.058	(0.000,	0.229)	(0.000,	0.240)	94	89.10	0	(0.000,	0.263)	(.,	.)
Distance visual acuity (ETDRS) 6 m	FILMS	127	60.24	0.175	(0.004,	0.327)	(0.013,	0.344)	NA						

BC 95% CI: Bias corrected bootstrapped 95% confidence interval; BCA 95% CI: Bias corrected and accelerated bootstrapped 95% confidence interval; ETDRS: Early Treatment Diabetic Retinopathy Study; EQ-5D: EUROQOL 5D-3L; HRQ: Hip replacement questionnaire; ICC: Intracluster correlation coefficient; m: month; min: minutes; NA: not applicable; Prop: proportion; RQLS: REFLUX quality of life measure; wk: week.

* 95% bootstrapped confidence interval using percentile method.

ICC estimates varied substantially between outcome type, though uncertainty around individual ICC estimates was substantial; this is reflected in generally wide confidence intervals. A summary of the ICC estimates by outcome is given in Table 3. Follow-up may also impact upon the ICC estimate as the largest values occurred when the outcome was measured closer in time to the intervention (Table 3). Most CIs were consistent with small or no clustering effect. There was evidence of a substantial clustering effect for some outcomes (e.g. operation time and length of stay). For others, there appeared to be little or no clustering (e.g. EQ-5D). ICC estimates appeared to be generally similar for surgeon and centre level clustering.

**Table 3:** Summary of ICC estimates

	Centre level				Surgeon level			
Outcome	N	Med	Min	Max	N	Med	Min	Max
*EQ-5D 1 wk*	1	0.029	-	-	1	0.023	-	-
*EQ-5D 3 m*	6	0.001	0	0.06	6	0.015	0	0.071
*EQ-5D 12 m*	5	0.007	0	0.04	5	0.002	0	0.04
*EQ-5D 60 m*	3	0.002	0	0.02	3	0.009	0	0.019
Oxford knee score *3 m*	3	0.041	0	0.07	3	0.05	0.007	0.068
Oxford knee score *12 m*	3	0.027	0.021	0.06	3	0.056	0.047	0.059
Oxford knee score *60 m*	3	0.044	0	0.05	3	0.037	0.002	0.051
*Operating time*	9	0.268	0.072	0.45	8	0.394	0.093	0.514
*Length of stay*	5	0.104	0.012	0.35	5	0.045	0	0.345
Surgical complications*	4	0.005	0	0.12	4	0.072	0.009	0.084
*HRQ overall score 4 m*	1	0.058	-	-	1	0.114	-	-
*HRQ overall score 12 m*	1	0	-	-	1	0	-	-
*HRQ overall score 24 m*	1	0	-	-	1	0	-	-
*RQLS score 3 m*	1	0.143	-	-	1	0.143	-	-
*RQLS score 12 m*	1	0.058	-	-	1	0	-	-
*Dist. visual acuity (ETDRS) 6 m*	1	0.175	-	-	-	-	-	-

*Dist.: distance; *EQ-5D: EUROQOL 5D-3L; ETDRS: Early Treatment Diabetic Retinopathy Study; HRQ: Hip replacement Questionnaire; ICC: Intracluster correlation coefficient; N: number of observations; m: month; Max: Maximum; Med: median; Min: minimum; RQLS: REFLUX quality of life measure; wk: week.

* Surgical complication group contains a variety of definition of complications and variable follow-up time periods.

Plausible impact on sample size under an expertise-based design and stratified within-surgeon design are shown in Table 4 for EQ-5D (12 months or longer) and operation time. For EQ-5D adoption of a stratified within-surgeon design protected against loss of information while the impact of an expertise-based trial design was dependent upon the anticipated cluster size if small (e.g. less than 10) the inflation of sample size was under 10%. However for large cluster sizes, as occurred in some of the trials, substantial increases in the required samples could be anticipated. For operation time, the large estimate ICC leads to large design effects even for very small average cluster size. Large design effects were plausible for an expertise-based trial design.

**Table 4:** Possible design effect for a continuous outcome based upon database

*Outcome*	*ICC†*	*Average cluster size*		Design effectχ	
				*expertise-based design*	*stratified design*
					
*EQ-5D 12 m*	0.01	min(centre)*	9.4	1.08	0.99
	0.01	median(centre)*	32.4	1.31	0.99
	0.01	max(centre)*	51.1	1.50	0.99
	0.01	median adj.(centre)‡	64.4	1.63	0.99
	0.01	min(surgeon)*	2.8	1.02	0.99
	0.01	median(surgeon)*	6.1	1.05	0.99
	0.01	max(surgeon)*	29.9	1.29	0.99
	0.01	median adj.(surgeon)‡	35.3	1.34	0.99
*Operation time*	0.27	min(centre)*	9.4	3.27	0.73
	0.27	median(centre)*	32.4	9.48	0.73
	0.27	max(centre)*	51.1	14.53	0.73
	0.27	median adj.(centre)‡	64.4	18.12	0.73
	0.27	min(surgeon)*	2.8	1.49	0.73
	0.27	median(surgeon)*	6.1	2.38	0.73
	0.27	max(surgeon)*	29.9	8.80	0.73
	0.27	median adj.(surgeon)‡	35.3	10.25	0.73

ICC: Intracluster correlation coefficient.

† ICC was based upon median of observed values.

* Average cluster size based upon actual cluster sizes across trials in the database.

‡ Average cluster size calculated using the formula (∑n_i_^2^)/∑n_i_) where n_i _is the number of observations in the i^th ^cluster.

χ Design effect was calculated using 1+(average cluster size-1)*ρ and 1-ρ for expertise-based trial and stratified design respectively.

The results of the three level multilevel model are shown in Table 5. The variance at the surgeon and centre levels appeared to be similar though the contribution of the surgeon level was slightly higher though there was a large amount of uncertainty regarding the relative proportioning of variance between these two levels. There was evidence of clustering when the variance of levels 2 and 3 were considered together.

**Table 5:** Three level multilevel model (patient within surgeon within centre)

*Outcome*	*ICC*	*Estimate^†^*	95% CI*	
*EQ-5D 12 m*	Level 2 (surgeon)	0.417	(0.000,	0.857)
	Level 3 (centre)	0.012	(0.000,	0.003)
	Levels 2 (surgeon) and 3 (centre)	0.029	(0.005,	0.053)

CI: confidence interval; EQ-5D: EUROQOL 5D-3L; ICC: Intracluster correlation coefficient.

***^†^***multilevel model using xtmixed command in Stata with no adjustment for treatment or other factors.

* 95% bootstrapped (bias corrected and accelerated) confidence interval

## Discussion

Our data on clustering effect for multicentre trials of surgical interventions suggests it is more of an issue than has been previously acknowledged. Despite the uncertainty intrinsic to estimating the magnitude of the ICC, there was evidence of clustering effect for a number of outcomes. As the outcomes with the highest ICC estimates (e.g. operation time and length of stay) are typically cost rather than clinical outcomes, clustering is likely to have the greatest impact on the economic evaluation. This database provides trialists with valuable information on how to design surgical trials. In particular it supports the wisdom of including either centre or surgeon (if a within-surgeon design is used) in the randomisation algorithm. The failure to analyse accordingly can result in a loss of precision [7,12].

The individual ICC estimates were suggestive of clustering for a number of outcomes. The ICC estimates for centre and surgeon level did not markedly differ as might be anticipated given that surgeons are typically nested within a centre. It is likely that the observed clustering by surgeon is driven by a number of factors and not just the surgeon per se. Furthermore where surgeon is used in the randomisation algorithm in practice this may function as a sub-centre (e.g. surgeons in the same surgical team) as opposed to reflecting an individual surgeon and hence can be in between centre and pure surgeon grouping. The latter is often more difficult to achieve than might be initially expected, particularly in a routine health care setting, as surgical trainees often undertake elements of the whole operation under the supervision of a senior surgeon or more senior surgeons work in a team environment.

The difficulties in estimating the uncertainty around a ICC estimate are well known [21]. We used the ANOVA method (along with bootstrapped confidence intervals) as it has been shown not to require any strict distributional assumptions and can be used for both continuous and binary outcomes [22]. Following other authors, we consider a negative ICC implausible; the ICC estimates were censored at zero [17,22]. Where the ICC estimate was close to zero, the reported ICC confidence interval limits may be slightly inflated as a consequence [15,17]. As surgical trial datasets do not tend to be large enough for precise estimate of ICC, the utilization of routinely collected data, perhaps in conjunction with surgical trial datasets, could be considered. Formal meta-analysis of ICC estimates would in principle provide the optimal use of available data and achieve greater precision [23]. Furthermore, ICC estimates can be calculated with adjustment for other important factors (e.g. baseline values for quality of life measures) which are likely to reduce the ICC estimates. Our estimates are unadjusted and therefore may be an overestimate of clustering provided the statistical analysis adjusts for such factors. The exploratory three level analysis suggested variance might be contributed from both the surgeon and centre levels as might be considered intuitively the case. However, even for the largest dataset in the database the uncertainty around the estimates was substantial.

Expertise-based trials have been used and promoted as a preferable design to the standard within-surgeon (stratified) design. Purported benefits of this design include increased surgical participation and compliance with randomisation, addressing the learning curve effect along with desirability from a patient perspective. However, expertise-based designs have been criticized on a number of grounds [24] including methodological considerations and particularly the required sample size. The data presented provides clarification on the potential impact which would appear to be related to the outcome(s) of interest. Expertise-based design, perhaps contrary to intuition, seems a (statistically) suboptimal choice for a comparison of surgical interventions where surgical outcomes (e.g. operation time, short recovery) are of interest. Of the trials included in the database, four focussed upon surgical primary outcomes. A better option would be surgeons with expertise in both surgical interventions delivering both interventions. In contrast an expertise-based trials seems a reasonable choice if long-term quality of life was the primary focus of the study as small, perhaps even zero, clustering was plausible for such outcomes. A caveat may be appropriate where stratification by centre was undertaken (and analysed accordingly) despite surgeons only delivering one or the other of the interventions. The impact of such an approach is unclear and empirical evaluation of statistical analysis options of an expertise-based trial is needed to evaluate the impact upon required sample size.

Where the clustering is anticipated to be small e.g. a longer term quality of life outcome, the potential recruitments benefits (particular of surgeons) of an expertise-based design could be seen as a reasonable offset to the loss of precision and need to recruit slightly more participants. It has been suggest that the effect size under an expertise-based design might be larger though there is little evidence to support such a premise at present. A hybrid [9] (or more specifically, surgeon preference) design, where surgeons are allowed to perform either one or both surgical interventions in a comparison of two surgical interventions, might therefore be the optimal design where the two surgical interventions substantially differ and the focus is on longer term quality of life outcomes.

Typically the statistical analysis of RCTs which allow for clustering across multicentre or therapists enable the underlying intercept level to vary between cluster but maintains a common treatment effect. Methods which allow for the treatment effect to vary between cluster in place of or in addition to underlying level has been proposed [25]. The relative impact of such options is unclear and further evaluation, specifically regarding the impact on sample size, is needed.

Differential clustering or clustering for only one intervention may be plausible. The method of analysis we undertook implicitly assumed a common ICC across the surgical interventions. For some of the settings represented by the surgical trials in our database (e.g. total knee arthroplasty with/without metal backed tibial component) a common ICC is very plausible where as for others (open versus laparoscopic hernia repair) this is perhaps less so. Due to the relatively small number of cases (as reflected in some CIs not being calculable) we choose to only calculate the common ICC across the interventions. This might be viewed as the most appropriate approach in the presence of any treatment effect.

There is a need for ICC estimates and providing data on cluster sizes to be routinely published [4,15]. This database of surgical ICC provides information to guide trialists in the design of trials evaluating surgical interventions as has been done for other areas [15]. Further research is needed into ICC estimates, both in their determinants and the optimal method of calculation (including consideration of meta-analysis). We anticipate that over time the addition of ICCs from further surgical trial datasets to our database will further inform the design of surgical trials; trialists are invited to submit surgical trial ICCs for inclusion in the database.

## Conclusions

Sizeable clustering effects in multicentre trials of surgical interventions at both centre and surgeon levels were plausible for some outcomes. A stratified design (by either centre or surgeon) with corresponding analysis provides optimal benefit with regard to sample size and protects against a potentially large loss of precision for surgical outcomes. Further research is needed into surgical ICCs, into both their determinants and the optimal method of calculation.

## List of abbreviations

**BC**: Bias corrected bootstrapped; **BCA**: Bias corrected and accelerated bootstrapped; **CI**: Confidence interval; **C-RCT**: Cluster randomised controlled trial; **EQ-5D**: EUROQOL 5D-3L; **ETDRS**: Early treatment diabetic retinopathy study; **HRQ**: Hip replacement questionnaire; **ICC**: Intracluster correlation coefficient; **M**: Month; **Min**: Minutes; **NA**: Not applicable; **Prop**: Proportion; **RCT**: Randomised controlled trial; **RQOL**: REFLUX quality of life measure; **WK**: Week

## Competing interests

The authors declare that they have no competing interests.

## Authors' contributions

JC obtained funding for the study and conceived of the study and conducted the analyses. TB, GM and CS contributed to the development of the study and/or to data management. All authors provided comments, read and approved the final manuscript.
